# Amino acid uptake in rust fungi

**DOI:** 10.3389/fpls.2015.00040

**Published:** 2015-02-05

**Authors:** Christine Struck

**Affiliations:** Group Crop Health, Faculty of Agricultural and Environmental Sciences, University of Rostock, Rostock, Germany

**Keywords:** biotrophy, haustorium, nitrogen, nutrition, plant pathogen

## Abstract

The plant pathogenic rust fungi colonize leaf tissue and feed off their host plants without killing them. Certain economically important species of different genera such as *Melampsora*, *Phakopsora*, *Puccinia*, or *Uromyces* are extensively studied for resolving the mechanisms of the obligate biotrophy. As obligate parasites rust fungi only can complete their life cycle on living hosts where they grow through the leaf tissue by developing an extended network of intercellular hyphae from which intracellular haustoria are differentiated. Haustoria are involved in key functions of the obligate biotrophic lifestyle: suppressing host defense responses and acquiring nutrients. This review provides a survey of rust fungi nitrogen nutrition with special emphasis on amino acid uptake. A variety of sequences of amino acid transporter genes of rust fungi have been published; however, transport activity of only three *in planta* highly up-regulated amino acid permeases have been characterized. Functional and immunohistochemical investigations have shown the specificity and localization of these transporters. Sequence data of various genome projects allowed identification of numerous rust amino acid transporter genes. An *in silico* analysis reveals that these genes can be classified into different transporter families. In addition, genetic and molecular data of amino acid transporters have provided new insights in the corresponding metabolic pathways.

## INTRODUCTION

Rust fungi (Basidiomycota, order: Pucciniales = Uredinales) are a group of more than 7000 plant parasites (Aime et al., 2006) causing diseases on many plants including most crops and ornamentals and are responsible for severe yield losses in numerous crops world-wide (Brown and Hovmøller, 2002). Most rust fungi are highly specialized pathogens and attack only certain genera, species, or even varieties of plants. Detailed information regarding our knowledge about rust fungi has been thoroughly described by Staples (2000) and Voegele et al. (2009).

Rust fungi are besides the ascomycetous powdery mildews, the downy mildews (stramenopiles, oomycetes) and the plasmodiophorids obligate biotrophic plant parasites. One major feature of biotrophic life style is extraction of nutrients from living host cells. Thus, the nature of biotrophy is characterized by an intimate relationship presenting the basis of the molecular cross talk between host cells and the biotrophic organisms. Some recent reviews address our current understanding of biotrophy. Yi and Valent (2013) give a comprehensive overview about the structures achieving the biotrophic plant cell colonization as revealed by light and electronic microscopical analyses. Rafiqi et al. (2012) summarized recent advances in identifying the so-called effectors, proteins secreted by the biotrophs and delivered to the host cells where they are involved in suppressing the defense response; and Spanu (2012) provides a comparative genomic approach to explain evolutionary aspects of fungal biotrophy.

Our knowledge about functional characteristics of nutrient uptake of biotrophic parasites is rather limited, e.g., carbohydrate uptake of *Uromyces fabae* is concentrated on the haustorial *HXT1*, a hexose transporter with specificity for glucose and fructose (Voegele et al., 2001). Up to now we have no evidences for any additional type of sugar transporter. However, the situation regarding uptake of nitrogen compounds seems to be more diverse. Rust fungi use a wide variety of N-sources, e.g., ammonia, amino acids and oligopeptides, as corresponding putative transporter genes, revealed by various genome sequencing projects, may indicate. However, these transportes have not been functionally characterized. The focus of this mini-review is based on earlier physiological and microscopical studies with recent genomic explorations of some model rust fungi.

## MECHANISM OF THE RUST FUNGAL NUTRIENT UPTAKE

### SPECIALIZED INFECTION STRUCTURES FOR AN EFFICIENT NUTRIENT UPTAKE

On the leaf surface rust spores produce germ tubes which grow on the plant surface. Over a stoma an appressorium is formed, a special structure from which the infection hypha is invading the leaf tissue (Hoch and Staples, 1987). Within the substomatal vesicle intercellular hyphae spread the plant tissue and haustorial mother cells are formed adjacent to mesophyll cells. Haustoria are formed after penetrating the plant cell wall without vulnerating the plant plasma membrane. The haustoria grow within the living plant cells and constitute an intimate contact to the plant cells cytoplasm. As a result the cytoplasm of host and fungus remain separated by the host plasma membrane, the fungal plasma membrane and between the so-called extrahaustorial matrix (Voegele et al., 2009). The position of haustoria within mesophyll cells has already in the nineteenths century encouraged the assumption that haustoria serve to the acquisition of nutrients from plant cells (de Bary, 1863).

### H^+^-ATPase FACILITATES NUTRIENT UPTAKE

The fact that haustoria are exclusively formed in plant tissue makes it rather difficult to unravel their function. Thus, results of early physiological and electron microscopical studies from the 1970s using radiolabeled metabolites (summarized by Mendgen, 1981), although giving indirect evidence for nutrient uptake by haustoria, were not found fully convincing. Only the isolation of rust haustoria (Hahn and Mendgen, 1992) combined with physiological analyses and applications of molecular genetic techniques reached more conclusive results. So, the activity of H^+^-ATPase in isolated haustorial plasma membrane of *U. fabae* (broad bean or faba bean rust) could be found strongly increased compared with the plasma membrane activity in uredospores and germ tubes (Struck et al., 1996). In addition, *PMA1* encoding a single copy H^+^-ATPase gene from *U. fabae* was isolated and functionally characterized by heterologous expression in yeast (Struck et al., 1998). These studies emphasized the expectation that the H^+^-ATPase plays a major role in parasitic growth of the rust fungus. The proton-motive force built up by the H^+^-ATPase might provide the energy for transport processes across the haustorial plasma membrane as well as across the plant plasma membrane, because in both, fungi and plants nutrient uptake is driven predominantly by an H^+^ electrochemical gradient. Thus, the rust H^+^-ATPase activity represents the basis for energizing nutrient transporters, including numerous amino acid transporters. The first evidence supporting this hypothesis was provided by identification of genes preferentially expressed in *U. fabae* haustoria and encoding putative nutrient transporters (Hahn and Mendgen, 1997; Hahn et al., 1997).

## CHARACTERIZATION OF AMINO ACID TRANSPORTERS OF RUST FUNGI

Uptake and translocation processes of nitrogen or nitrogenous compounds are of fundamental importance for all life as the element nitrogen is a basic component of proteins and nucleic acids. Therefore, understanding of molecular aspects on N acquisition systems is essential. Numerous fungal transporters capable of taking up amino acids have been described (Wipf et al., 2002b) and classified in the Transporter Classification Database (TCDB, Saier et al., 2013). Based on sequence analyses most of them can be classified as members of the APC (amino acid polyamine choline, 2.A.3.) superfamily, which show a wide range of substrate specificities. Members share a common topology, comprising of 12 putative membrane-spanning domains and they carry out amino acid/H^+^-symport (Jack et al., 2000; Yen et al., 2009).

Up to now, three amino acid transporter genes of rust fungi and their corresponding proteins have been characterized in more detail. First evidence for an amino acid permease was based on a *U. fabae* haustorium-specific cDNA library (Hahn and Mendgen, 1997), which revealed, among others, two genes (*PIG2* and *PIG27*) encoding proteins with high similarity to fungal amino acid transporters. Later these *in **p**lanta* **i**nduced **g**enes were renamed to Uf-*AAT2* and Uf-*AAT1* (Mendgen et al., 2000) and another gene (Uf-*AAT3*) encoding an amino acid permease has been isolated from *U. fabae* (Struck et al., 2004).

Sequence analyses revealed that these three genes are transmembrane proteins belonging to the fungal specific YAT family (TC 2.A.3.10, yeast amino acid transporter), a member of the above mentioned APC superfamily. Expression analyses showed that amino acid transporters of *U. fabae* are developmentally regulated in different ways. Whereas AAT1 and AAT3 are expressed very early during rust development and are strongly up-regulated in haustoria (Struck et al., 2002, 2004), AAT2 was shown strictly haustorium specific. With antibodies raised against synthetic AAT2 peptides, the *AAT2*-encoded protein was localized exclusively to plasma membranes of the haustorial bodies (Hahn et al., 1997).

Whereas, substrate specificity of the strictly haustorium specific AAT2 amino acid permease have not been enlightened until now, substrate specificity for *U. fabae*-AAT1p and AAT3p could be clarified. Functional complementation and uptake experiments in a *Saccharomyces cerevisiae* strain defective in biosynthesis and uptake of histidine revealed that AAT1p is able to transport a series of amino acids whereby highest activities were obtained with histidine and lysine. In addition, heterologous gene expression in *Xenopus laevis* oocytes revealed AAT1p-dependent symport of a broad spectrum of amino acids (Struck et al., 2002). In contrast, electrophysiological experiments using AAT3p-injected oocytes showed substrate preferences for leucine and the sulfur containing amino acids methionine and cysteine (Struck et al., 2004). Summarized, transcripts of both *U. fabae*-*AAT1* and *Uf* - *AAT3* were found to encode for amino acid permeases with more or less broad substrate specificity and are present throughout parasitic phase within the host plants with accumulation to the highest level in haustoria.

## NOVEL INSIGHTS FROM GENOMIC APPROACHES

Sequence based methods of analyzing genes have been expanded tremendously. The increasing number of genomic resources enables us to identify both species unique genes as well as functionally homologous genes of interesting phenotypes. Furthermore, the huge influx of data from genetic and comparative genomics approaches may contribute to elucidate the general traits of biotrophic life style of economically important crop pathogens (reviewed by Spanu, 2012). For example, analysis of the haustorial cDNA library of the powdery mildew fungus *Golovinomyces orontii* infecting *Arabidopsis thaliana* has shown that transcripts encoding putative nutrient transporters were not as highly represented in haustoria of powdery mildew as in rust fungal haustoria (Weßling et al., 2012). Based on pathogen genomes Kemen and Jones (2012) presented hypotheses of general patterns of genome evolution of filamentous fungi and oomycetes to explain parasitic life styles.

Another approach presented Chibucos and Tyler (2009) when describing how to use the gene ontology database by using terms developed by the Plant-Associated Microbe Gene Ontology (PAMGO) Consortium. They analyzed the acquisition of nutrients of plant symbionts and identified commonalities in symbiotic nutritional strategies among diverse plant symbionts, which includes a comparison between pathogenic and mycorrhizal fungi. High expression of amino acid transporter genes have been documented in both the extraradical mycelium and the intraradical mycelium of the arbuscular mycorrhizal fungus *Rhizophagus irregularis* (formerly *Glomus intraradices*) suggesting that the fungal symbiont can take up amino acids from the soil as well from the plant apoplast (Tisserant et al., 2012).

### *IN SILICO* COMPARISON OF AMINO ACID TRANSPORTERS

**FIGURE 1:**
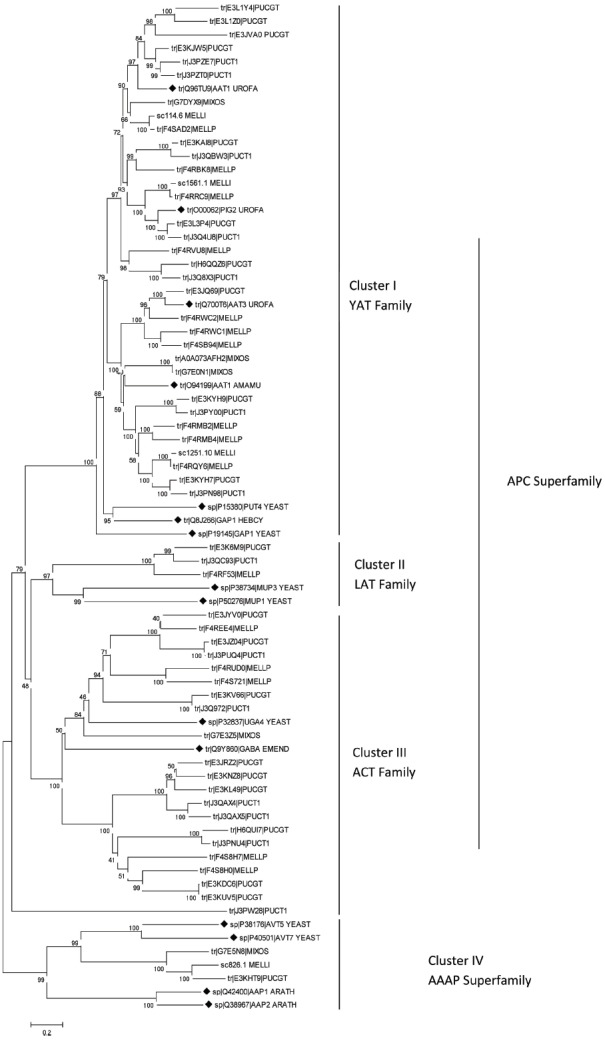
**Neighbor joining tree of (putative) amino acid transporters of rust fungi based on full-length amino acid sequences.** Bootstrap values indicated next to branches are from 1000 replications. The formerly characterized transporters are labelled by black diamonds. Sequences are designated with Uniprot ID and five letter species names: ARATH, *Arabidopsis thaliana*; EMEND, *Emericella nidulans*; HEBCY, *Hebeloma cylindrosporum*; MELLI, *Melampsora lini*; MELLP, *M. larici-populina*; MIXOS, *Mixia osmundae*; PUCGT, *Puccinia graminis-tritici*; PUCT1, *P. triticina*; UROFA, *Uromyces fabae*; YEAST, *Saccharomyces cerevisiae*.

In the past 5 years sequencing projects of several rust fungi genomes has been performed. Thus, our knowledge about the existence of amino acid transporters in rust fungi comes from various species: the important cereal rusts *Puccinia graminis* (Duplessis et al., 2011a), *Puccinia striiformis* (Garnica et al., 2013) and *Puccinia triticina* (Zhang et al., 2008), the poplar leaf rust *Melampsora larici-populina* (Hacquard et al., 2010; Duplessis et al., 2011b), the flax rust *Melampsora lini* (Nemri et al., 2014), the coffee leaf rust *Hemileia vastatrix* (Vieira et al., 2012; Talhinhas et al., 2014) and the unusual fern rust *Mixia osmundae* (Toome et al., 2014). *In silico* analyses comparing the sequences of putative amino acid transporters of rust fungi with some functionally well characterized fungal amino acid permeases which belong to different transporter families were conducted and uncovered the presence of distinct groups of AATs (Figure 1). As members of the YAT family the ascomycetous *S. cerevisiae* PUT4 protein (Vandenbol et al., 1989) has been selected together with the only basidiomycetous characterized amino acid transporters: the ectomycorrhizal permeases GAP1 of *Hebeloma cylindrosporum* (Wipf et al., 2002a) and AAT1 of *Amanita muscaria* (Nehls et al., 1999) and the above mentioned *U. fabae* amino acid permeases AAT1, AAT2 (synonym PIG2), and AAT3 (Hahn et al., 1997; Struck et al., 2002, 2004). The yeast methionine specific transporters MUP1 and MUP3 (Isnard et al., 1996) were selected as members of the LAT family (L-type amino acid transporter, 2.A.3.8). The γ-aminobutyric acid transporters UGA4 of *S. cerevisiae* (André et al., 1993) and gabA of *Emericella nidulans* (Hutchings et al., 1999) were selected as members of the ACT family (amino acid/choline transporter, 2.A.3.4). From the AAAP superfamily (amino acid/auxin permease) the vacuole amino acid transporters of *S. cerevisiae* AVT5 and AVT7 (Russnak et al., 2001; Tone et al., 2014) have been selected and the *Arabidopsis thaliana* permeases AAP1 and AAP2 (Kwart et al., 1993) as outgroup. The Universal Protein Resource database (www.UniProt.org) was searched for full length protein amino acid sequences of putative rust fungal amino acid transporters. Sequence fragments were excluded. In total 60 transporter proteins of *P. graminis-tritici* (21), *P. triticina* (14), *M. lini* (4), *M. larici-populina* (16), the fern rust fungus *Mixia osmundae* (5) and the 13 above mentioned characterized proteins were aligned using ClustalW. The phylogenetic analyses were performed using the Neighbor-Joining method (Saitou and Nei, 1987) implemented in MEGA6.0 (Tamura et al., 2013) using the Poisson correction model (Zuckerkandl and Pauling, 1965) and pairwise deletion of gaps option for distance computation.

Phylogenetic reconstruction (Figure 1) reveals that the vast majority of the putative amino acid transporters belong to the APC superfamily; however, they cluster into three different transporter families. Thirty three of the 60 rust fungal AAT sequences cluster with the YAT family, 20 sequences cluster with the ACT family and three cluster with the methionine specific LAT family. Most of the well characterized fungal AATs belong to the YAT family including GAP1, PUT4, and numerous others. Physiological analyses have not only revealed the transport function but also the sensor function of certain AATs, which show only low transport activity. In *S. cerevisiae* the YAT member Ssy1 is known to sense extracellular amino acids and induce the expression of the corresponding amino acid permeases (Didion et al., 1998; Forsberg and Ljungdahl, 2001). Recently, Aouida et al. (2013) have revealed that the plasma membrane protein Agp2 is not only involved in uptake of L-carnitine and polyamine but is also responsible for regulation of expression of polyamine uptake transporters.

One putative ortholog of the yeast vacuolar amino acid transporters (AVTs) from each of the genus *Mixia*, *Melampsora*, and *Puccinia* have been found; however, we have to assume that rust species differ in the number of AATs they contain. For example, Talhinhas et al. (2014) have found three genes of the coffee rust *Hemileia vastatrix* belonging to the AAAP superfamily. Nevertheless, the vacuolar transporters certainly do not play a central role in nitrogen nutrition and amino acid uptake of fungi.

In conclusion, although it might be possible to infer the function of the proteins from their position in the phylogenetic tree, functional analyses of the putative transporters are needed before drawing reliable conclusions about their physiological activity.

### INDIRECT INSIGHTS IN REGULATORY PROCESSES

Transcript allocation and expression analyses make it possible to predict metabolic pathways. Various transporter genes of rust fungi have been identified and expression patterns showed that they are distributed on haustoria and in intercellular hyphae and to a lesser extend in spores and germ tubes. Based on genome projects of *M. larici-populina*, *P. striiformis*, and *M. lini* it could be hypothesized that no functional nitrate assimilation pathway exists in rust fungi as the corresponding genes encoding a nitrate reductase or nitrite reductase appeared to be expressed at an extremely low level in infected tissue or could not be identified (Duplessis et al., 2011a; Garnica et al., 2013; Nemri et al., 2014). Thus, it is assumed that nitrate and nitrite transporters are not relevant for nutrition of these rust species. Until now we have—with the exception of the *U. fabae* amino acid permeases, no functional evidence for enzymes involved in amino acid metabolism. Analyses of sets of haustorial genes of a number of important rust fungi do not allow conclusive statements about the haustorial metabolism. By sequencing the *P. striiformis* f. sp. *tritici* transcriptome Garnica et al. (2013) also identified a gene that encodes a transporter with very high similarity to a S-methylmethionine permease, which was almost exclusively expressed in haustoria compared to germinated spores and might be responsible for uptake of sulfur compounds. However, genes encoding enzymes involved in amino acid biosynthesis and metabolism have been identified (Jakupović et al., 2006; Garnica et al., 2013) suggesting ammonia and amino acids from the host plants can be metabolized.

## FUTURE PERSPECTIVES

During the next years it is to be expected that a growing number of fungal genomes will be available and will give a more clear insight also to rust fungal biotrophic nutrient acquisition. However, up to now our knowledge is restricted to transporters more or less highly expressed in different stages of rust fungi which lead to the assumption that these are the vital nutrient transporters. Future studies should unravel the complete inventory of transporters relevant for nitrogen uptake. Also, efforts need to detect both factors involved in nutrient signaling and regulatory elements of amino acid uptake of rust fungi. Thus, what are needed are additional functional studies of the predicted proteins involved in haustorial metabolism. Such elements could enable us to understand how plant pathogens have adapted to their host plants. In addition, it might be highly interesting to compare the repertoire of AATs of obligate biotrophic organisms of different systematic position with each other, e.g., the ascomycete *Blumeria graminis*, the glomeromycete *R. irregularis* and representatives of protists such as the oomycete *Albugo* sp. to get more detailed insights in the physiological and molecular basis of biotrophic life style and differences between parasitism and mutualism.

### Conflict of Interest Statement

The author declares that the research was conducted in the absence of any commercial or financial relationships that could be construed as a potential conflict of interest.
